# Co-cultured Bone-marrow Derived and Tendon Stem Cells: Novel Seed Cells for Bone Regeneration

**DOI:** 10.1515/biol-2019-0063

**Published:** 2019-12-31

**Authors:** Yang Liu, Chengsong Yuan, Mei Zhou, Kanglai Tang

**Affiliations:** 1 Department of Orthopaedics, First Affiliated Hospital, Army Military Medical University, Chongqing 400038, P.R. China; 2 Department of Orthopaedics, First Affiliated Hospital, Army Medical University, Chongqing, P.R. China 400038

**Keywords:** Tenascin-C, Bone-marrow derived stem cells, Tendon stem cells, Co-culture

## Abstract

Tendon-bone healing after injury is an unsolved problem. Several types of stem cells are used as seed cells. However, the optimal co-culture ratio of different types of cells suitable for tissue engineering as well as the stimulator for facilitating the differentiation of stem cells in tendon-bone healing is unclear. In this study, the proliferation of both bone marrow-derived stem cells (BMSCs) and tendon stem cells (TSCs) was increased at a 1:1 co-cultured ratio, and proliferation was suppressed by Tenascin C (TNC). TNC treatment can promote osteogenesis or chondrogenesis of both BMSCs and TSCs under a 1:1 co-cultured ratio. In addition, the expression level of Rho-associated kinase (ROCK) increased in the process of TNC-induced osteogenesis and decreased in the process of TNC-induced chondrogenesis. Furthermore, the level of insulin-like growth factor 1 receptor (IGF-1R) and mitogen-activated protein kinase (MEK) was upregulated during the osteogenesis and chondrogenesis of both BMSCs and TSCs after TNC treatment. Although our study was conducted in rats with no direct evaluation of the resulting cells for tendon-bone healing and regeneration, we show that the proliferation of BMSCs and TSCs was enhanced under a 1:1 co-cultured ratio. TNC has a significant impact on the proliferation and differentiation of co-cultured BMSCs and TSCs. IGF-IR, ROCK, and MEK may become involved in the process after TNC treatment.

## 1 Introduction

Enthesis injuries result in great suffering for both athletes and ordinary people [1]. The capacity of enthesis self-healing is low, scar formation is the primary repair process, and this results in inferior mechanical load-handling of interfaces and leads to re-injury [2]. The complexity of tissue components involved in enthesis is one of the most important causes of for this poor regenerative ability. Under normal conditions, well-organized tendon, cartilage, and bone create a complex stratified tissue that currently cannot be fully recovered after injury. Within this complexity, cartilage is the most critical component and is responsible for a trasition from soft tissue to hard tissue. However, insufficient cells in this area raise the difficulty of cartilage regeneration, partly because the original cellular source of chondrogenesis in this area still remains unclear.

Stem cells are considered as a reasonable source for tissue repair since they can differentiate into various mature cells [2, 3, 4]. Considering that the fibrocartilaginous region is avascular, the endogenous progenitor cells which reconstitute function would be derived from the surrounding zone, including tendon, bone, or hematoma following tissue injuries. As the most promising and safe source for cell therapeutic applications, bone marrow-derived stem cells (BMSCs) are widely used in clinical applications [5] and contribute to enthesis healing [6]. On the other hand, tendon stem cells (TSCs) have recently been identified within tendon tissues and might be an effective source for enthesis regeneration due to their universal stem cell characteristics, such as clonogenicity, a high proliferative capacity, multi-differentiation potential, non-immunogenicity, and immunosuppression [7, 8]. On the basis of these features, BMSCs or TSCs can be considered as suitable candidates used for enthesis repair in tissue engineering. Furthermore, previous studies have indicated that use of two or more cell populations has been attempted to

evade limitations of monoculture systems and promote or induce chondrogenesis [9, 10], but there are still some questions to be solved including the optimal ratio of BMSCs and TSCs that will promote the differentiation to desired tissue [11,12].

Besides stem cells, growth factors are useful for aiding cell differentiation in tendon tissue engineering. Pro-tenogenic growth factors are peptide signaling molecules with a dominant biological role in regulating cell proliferation and differentiation [13]. Growth factors relevant to the tendon healing process and MSC tenogenesis include the bone morphogenetic protein (BMP), fibroblast growth factor (FGF), transforming growth factor beta (TGF-β), insulin-like growth factor (IGF), vascular endothelial growth factor (VEGF), connective tissue growth factor (CTGF) and platelet-derived growth factor (PDGF) families [12]. Moreover, extracellular matrix (ECM) protein also has been thought as a tenogenic growth factor. Tenascin-C (TNC) as a member of the hexabrachion-shaped ECM protein family has restricted expression in normal musculoskeletal tissues, while the expression of TNC is significantly increased during embryogenesis or regenerative /healing processes [14]. TNC is present in all musculoskeletal regions in which high mechanical forces are transmitted from one tissue component to another, for example from muscle to tendon and from tendon to bone [15, 16]. Whether or not TNC could promote osteogenesis and chondrogenesis is still unclear.

Our present study addresses the favorable ratio of TSCs and BMSCs co-cultured for chondrogenesis, pointing out the potential ability of TSCs to differentiate into chondrocytes. Furthermore, treatment with TNC can impact the proliferation and differentiation of co-cultured stem cells. In addition, IGF-IR, ROCK, and MEK are involved in TNC-promoted BMSC and TSC differentiation. This research provides insight into the efficient selection of seed cells for cartilage repair in tissue engineering.

## 2 Materials and Methods

### 2.1 Isolation and Culture of BMSCs and TSCs

Six 8-week-old male Sprague–Dawley (SD) rats (250–300 g) were used to isolate stem cells. All experiments were approved by the Animal Research Ethics Committee, Army Medical University, China. The harvesting of BMSCs and TSCs from the rats and their culture were carried out as described previously [17, 18]. Briefly, the processes are described below. For TSC isolation and culture, a whole piece of intact Achilles’s tendon was excised from both limbs after sacrificing each rat. Only the mid-substance tissue was collected, and the surrounding paratenon was carefully removed. Then the tissues were cut into small pieces and digested for 1 h at 37℃ with type I collagenase (3 mg/ml Sigma–Aldrich, St. Louis, MO, USA) and dispase II (4 mg/ml) (Gibico, USA). The tissue enzyme solution was filtered with a 100-μm cell strainer. Single-cell suspensions were washed twice with sterile phosphate-buffered saline (PBS) followed by centrifugation at 300 ×g for 5 min and resuspended in Dulbecco’s modified Eagle’s medium (DMEM; Gibco, Carlsbad, CA, USA) with 10% fetal bovine serum, 100 U/ml penicillin, 100 mg/ml streptomycin, and 2 mM L-glutamine (all from Invitrogen, Carlsbad, CA, USA). The isolated cells were diluted to 500 cells/cm^2^ and cultured at 37℃ in 5% CO_2_ to form colonies. After 2-day initial plating, the cells were then washed twice with PBS to remove nonadherent cells. At day 7, cells were trypsinized and mixed together.

The BMSCs were isolated from rat femurs and tibiae. Marrow was harvested by inserting a 20-gauge syringe needle and flushing with MSCs growth medium (Cyagen Biosciences, Sunnyvale, CA), followed by filtration with a 70-μm cell strainer. Cells (500 cells/cm^2^) were inoculated in a flask, cultured in a humidified incubator containing 5% CO_2_ at 37°C, and allowed to attach for 48 h. Nonadherent cells were removed by changing the culture medium. Subsequent medium would be changed every 2-3 days. Subculturing was performed when the primary cells became 80% confluent.

**Ethical approval:** The research related to human use has been complied with all the relevant national regulations, institutional policies and in accordance the tenets of the Helsinki Declaration, and has been approved by the authors’ institutional review board or equivalent committee.

### 2.2 Immunofluorescent staining

The isolated BMSCs and TSCs, grown on coverslips, were fixed with 4% paraformaldehyde at room temperature for 30 min. After being washed twice with PBS and incubated with a permeabilization solution (0.1% Triton X-100) for 10min, the cells were incubated with the CD44 or CD90 antibody (1:50, Abcam, Cambridge, UK) at 4 ℃ for 12 hrs, followed by Cy3 or FITC-conjugated goat anti-rabbit IgG antibody (1:1000 dilution, green; Jackson ImmunoResearch Laboratory, West Grove, PA), and DAPI for 5min. Immunofluorescence images were acquired withlaser confocal microscopy (Olympus AX70).

### 2.3 Co-culture condition

Direct or indirect co-culture of BMSCs and TSCs was performed as described by previous studies [19, 20]. To identify the ideal cell ratio in direct contact co-culture systems, five different cell ratios (BMSCs/TSCs: 1:1, 1:3, 2:3) were modulated. And the effects of indirect co-culture was studied using a transwell co-culture system. BMSCs (2 × 10^4^ cells per well) were seeded into the upper compartment of a six-well transwell system (8 μm pore polycarbonate membrane). TSC cells were placed into the lower compartment of the six-well transwell system (2 × 10^4^ cells per well). All of the cells were cultured at 37℃ in 95% air/5% CO_2_ atmosphere in Dulbecco’s Modified Eagle’s Medium (DMEM)/F12 containing 10% fetal bovine serum. Three days after TNC or three inhibitor treatment, cells were harvested for real-time polymerase chain reaction (RT-PCR) analysis or other further studies.

### 2.4 Cell proliferation and migration assay

Cell proliferation was assessed by cell counting kit-8 (CCK-8, Beyotime, Shanghai, China). Cells from different BMSC/TSC ratios with or without TNC (1 to 100 μg/ mL, recombinant human tenascin c protein, Abcam, Cambridge, UK) treatment were seeded in 96-well plates. The optical density (OD) values of cells at absorption 460 nm were measured at day 1, 2, 3, or 7 according to the manufacturer’s instructions by spectrophotometer (Bio-Tek ELX800, USA).

### 2.5 RNA extraction and RT-PCR

RNA was extracted with Trizol and quantified by Nano-Drop spectrophotometer. After synthesizing cDNA, the peroxisome proliferator-activated receptor γ (PPARγ), runt-related transcription factor 2 (RUNX2), Scleraxis (SCX), and Sry related HMG box gene 9 (SOX-9) were amplified by quantitative Real-time PCR via a SYBR green premix according to the manufacturer’s guide (Qiagen, Hilden, Germany). Relative expression of target genes was standardized to GAPDH, evaluated by the 2^-ΔΔCT^ method, and given as a ratio as a control. The primer information is listed in Table 1.

**Table 1:** Primers for PCR

Gene	Primer（5’- 3’）	
SCX	F	GCAAGCTCTCCAAGATTGAG
	R	CGTCTTTCTGTCACGGTCTT
PPAR **γ**	F	CCTTTACCACGGTTGATTTCTC
	R	GGCTCTACTTTGATCGCACTTT
GAPDH	F	TGACTTCAACAGCAACTC
	R	TGTAGCCATATTCATTGTCA
RUNX2	F	GAACTCAGCACCAAGTCCTTT
	R	CAGTGTCATCATCTGAAATACGC
SOX-9	F	GCCCCTTCAACCTTCCGCACTAC
	R	CGGCTGCGTGGCTGTAGTAGGA

F=forward; R=reverse; GAPDH=glyceraldehyde phosphate-3 dehydrogenase (internal control).

### 2.6 Immunoblotting

Cultured BMSCs and TSCs were lysed in RIPA lysis buffer (Beyotime, Shanghai, China). The homogenates (20 mg of protein) were separated by 8% SDS-polyacrylamide gel electrophoresis and transferred onto polyvinylidene fluoride (PVDF) membranes. The blots were then washed with Tris-buffered saline Tween-20 (TBST), blocked with 1% BSA in TBST buffer for 1 hr, and incubated with the RUNX2 and SOX-9 antibodies (1:1000, Cell Signaling Technology, MA). Then, the primary antibodies were detected with goat anti-rabbit-IgG (1:5000, Jackson ImmunoResearch Laboratory, PA）conjugated to horseradish peroxidase, and the bands were visualized with enhanced chemiluminescence (Pierce, MA). The amount of protein transferred onto the membranes was verified by immunoblotting for GAPDH (1:500, Santa Cruz, CA).

### 2.7 Statistical analysis

Data were expressed as mean ± SD and analyzed by SPSS 19.0 (SPSS Inc, Chicago, IL, USA). Independent sample t tests were used to compare means in two different groups. One-way analysis of variance (ANOVA) was used to compare the data from more than two groups. A value of P <0.05 was considered as statistically significant.

## 3 Results

### 3.1 The surface markers of BMSCs and TSCs

**Fig 1:**
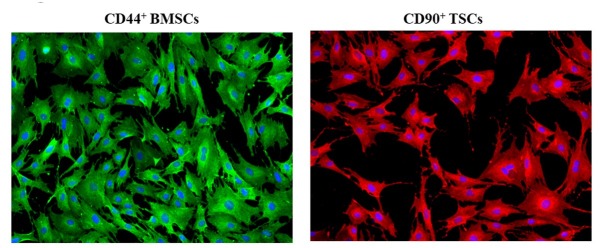
**Identifying BMSCs and TSCs.** CD44 (green) and CD90 (red) were verified by immunofluorescent staining. Experiments were repeated at least three times.

To verify the isolation of BMSCs and TSCs, the surface biomarkers of BMSCs and TSCs were visualized via immunofluorescent staining, and the data showed that CD44, one of the surface markers of BMSCs, and CD90, one main surface marker of TSCs, were expressed positively, indicating that the isolated cells were BMSCs and TSCs (**Fig. 1**).

### 3.2 Effect of BMSC and TSC co-culture and TNC treatment on BMSC and TSC proliferation

**Fig 2:**
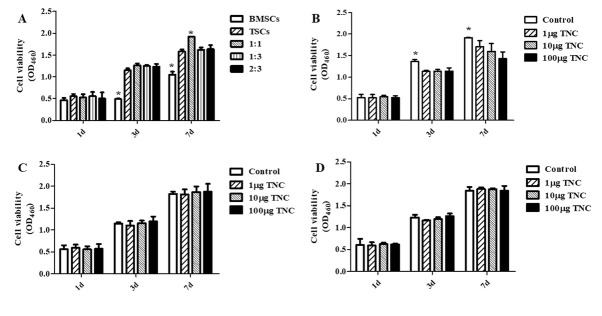
**The effect of BMSC/TSC co-culture (A) and TNC treatment (B to D) on cell proliferation.** CCK-8 assay was performed to detect cell proliferation. The effect of TNC on different co-culture ratios 1:1 (**B**), 1:3 (**C**) and 2:3 (**D**) was analyzed. (*P<0.05 vs. other groups in the same day, n=6).

To assess the proliferative ability of BMSCs and TSCs under direct co-culture and the effect of TNC on them, a CCK-8 assay was performed. The data found that the BMSC proliferation rate was significantly lower than that of TSCs and co-cultured cells after 3 to 7-day culture. Moreover, the 1:1 BMSC/TSC co-culture led to increased cell proliferation after 7 days, as compared with other groups (**Fig. 2A**). Since TNC is involved in the development of tendons, bone, and cartilage [21], we then assessed the role of TNC in cell proliferation, and the results showed that TNC treatment (1-100 μg/ml) decreased the proliferation of 1:1 co-cultured BMSCs/TSCs (**Fig. 2B**). And TNC did not show any effect on the proliferation rate of BMSCs/TSCs with other co-culture ratios (**Fig. 2C** and **D**).

### 3.3 Effect of BMSC and TSC co-culture or TNC treatment on differentiation

**Fig 3:**
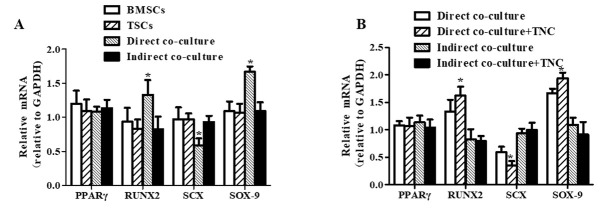
**The detection of differentiation-associated gene expression.** The differentiation-associated genes, including PPARγ, RUNX2, SCX and SOX-9 were analyzed by RT-PCR. (*P<0.05 vs. other groups, n=6).

To investigate adipogenesis, osteogenesis, tenogenesis, and chondrogenesis of BMSCs and TSCs, gene marker expression was tested by RT-PCR. We found that direct co-culture significantly increased expression of RUNX2, a regulator of osteoblast cell cycle entry, and SOX-9, a key transcription factor in chondrocyte differentiation; whereas SCX, a marker of tendon tissue formation, was decreased in the direct co-culture model, and indirect cell co-culture did not affect the expression of any gene markers (**Fig. 3A**). Furthermore, TNC significantly increased RUNX2 and SOX-9 gene expression and decreased SCX gene expression in the direct co-culture model, but it did not affect the expression of any gene markers in an indirect cell co-culture model (**Fig.3B**).

### 3.4 ROCK, IGF-IR, and MEK are involved in TNC-induced promotion of BMSC and TSC differentiation

**Fig 4:**
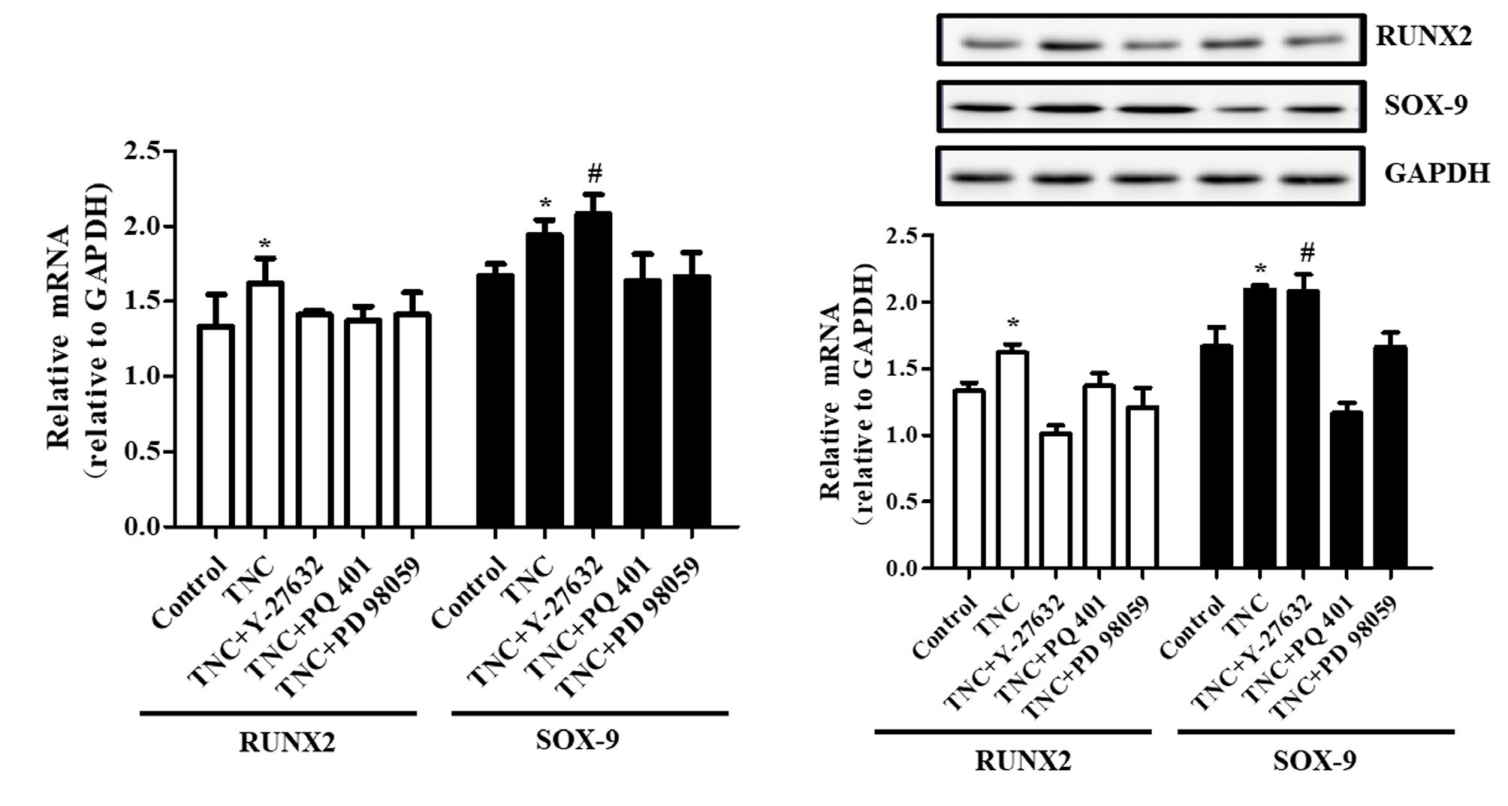
**The effect of signaling protein inhibitors.** RUNX2 and SOX-9 gene (**A**) and protein (**B**) expression were detected by RT-PCR and immunoblotting. (*P<0.05 vs. control and TNC+ Y-27632/PQ 401/PD 98059, ^#^P<0.05 vs. control, TNC group and TNC+ PQ 401/PD 98059, n=6).

We next analyzed the gene expression and signaling of BMSC and TSC differentiation induced by TNC. At first, Y-27632, a Rho-associated protein kinase (ROCK) inhibitor (30 μmol/L, Sigma, USA), was used to analyze the role of ROCK in the expression of the differentiation-associated gene markers RUNX2 and SOX-9. And the data shows that Y-27632 decreased TNC-induced RUNX2 gene and protein expression and increased SOX-9 gene and protein expression. Besides, PQ 401, an IGF-1R inhibitor (1 μg/ml, Abcam, USA), decreased TNC-induced RUNX2 and SOX-9 gene and protein expression. And MEK inhibition by PD 98059 (50 μmol/L, Target Mol, USA) significantly reduced the increased RUNX2 and SOX-9 gene and protein expression following TNC treatment (**Fig. 4**).

## 4 Discussion

Due to the complexity of enthesis, regeneration following injury is difficult, partly because the components, which include tendon, cartilage, and bone, have low regenerative ability. Under these circumstances, stem cells are considered to be proper candidates for enthesis recovery. BMSCs and TSCs are two major candidate cell sources that have been exploited for their tissue-regenerative properties and used in tendon tissue engineering and cell therapy [17, 18, 19, 20]. In the present study, we tested different ratios of co-culture and found that a 1:1 co-culture ratio of BMSCs and TSCs can promote proliferation comparedto BMSCs or TSCs alone. Interestingly, this feature seems to only be effective under a 1:1 ratio while ratios of 1:3 or 2:3 have no similar effect. The indicated interaction between different types of cells can be impacted by different ratios, suggesting that the ratio of co-cultured seed cells is a critical factor to be considered in tissue engineering. In regard to differentiation, we found 1:1 co-cultured BMSCs/TSCs produced to the markers of osteogenesis or chondrogenesis, i.e. RUNX2 and SOX9, up-regulated significantly compared to individual cell culture or indirect co-culture. The results showed that differentiation can be enhanced by direct contact of BMSCs and TSCs but not by indirect contact. Interestingly, tenogenesis can be suppressed under direct co-culture. These findings imply that TSCs and/or BMSCs tend to differentiate into cartilage or bone but not tendon, which may be beneficial to enthesis repair [22].

Growth factors are key factors in tendon tissue engineering and in regulating cell proliferation and differentiation, which lead to the tendon healing process [13]. Furthermore, BMSCs have been found to be very sensitive to growth factors which influence their stemness and steer the rate of proliferation and extent of terminal differentiation [23, 24]. TNC is an extracellular matrix protein upregulated during tissue remodeling [25]. De Laporte *et al*. found that TNC binds growth factors via its fourth and principally fifth fibronectin type III-like domains with nanomolar affinity [26]. TNC III 1–5 was expressed and shown to bind a wide variety of growth factors from different families including the PDGF family, the FGF family, the TGF-β superfamily, and neurotrophins, in addition to the IGF-BPs. This promiscuous affinity for growth factors from diverse families may be related to the broad role of TNC in tissue repair.

Moreover, previous studies showed that TNC inhibits cell spreading by binding to the XIII^th^ fibronectin-type III repeat of fibronectin [27, 28]. Consequently, the activities of RhoA and focal adhesion kinase are compromised: cells redistribute their actin to the cell cortex and downregulate focal adhesion formation [29, 30]. Thus, our data show that TNC suppresses co-cultured BMSC and TSC proliferation which may be associated with the regeneration of the fibrocartilage layer of the tendon-bone interface. Our study also found that TNC treatment leads to osteoblast or chondrocyte differentiation in directly co-cultured cells but not in the indirectly co-cultured cells, indicating that cell-cell junctions might play a key role in TNC-induced osteoblast and chondrocyte differentiation.

Furthermore, our study analyzed the signaling of BMSC and TSC differentiation induced by TNC. Our results showed that ROCK increased TNC-induced osteocyte differentiation and decreased TNC-induced chondrocyte differentiation. As a major downstream effector of the small Rho GTPases, ROCK regulates gene expression [31] and differentiation and proliferation of several types of stem cells [32, 33]. Inhibition of ROCK can regulate the ability of stem cells to differentiate into derivatives of all three germ layers [34]. We also determined that IGF-1R up-regulates TNC-induced osteogenesis or chondrogenesis. IGF-1R is a transmembrane tetramer receptor that exists as heterodimers composed of two α and β hemireceptors linked by disulfide bonds in a β–α–α–β structure [35]. After ligand binding, the downstream signaling of IGF-1R is dependent on the differential phosphorylation pattern of its β-subunit and the resultant tyrosine residues available to initiate mitogenic signals, possibly through the extracellular signal-related kinase (ERK) [36]. IGF-1R has been determined to induce transcriptional activity that promotes the differentiation of MSCs [37]. Lund AW *et al*. found that the inhibition of ERK alters cell-mediated proliferation, followed by development of distinct tissue microstructure, suggesting that the ability to reorganize collagen in 3D is an important step in ERK-mediated osteogenic differentiation [33]. MEK activates ERK. Our data also suggest that MEK increases TNC-induced osteocyte and chondrocyte differentiation, which might be dependent on the ERK/IGF-I pathway.

The current study has several limitations. Although we explore a potential approach to increase BMSCs and TSCs initially, fully understanding the clinical significance still requires further animal experimentation and human studies. Since traditional enthesis is a gradual process with spatial gradients that minimize stress concentrations and mediate load transfer between the soft and hard tissues, co-culturing BMSCs and TSCs might partly address enthesis injury repair. Our further research will focus on this topic.

In conclusion, our studies indicate that a 1:1 ratio of co-cultured BMSCs and TSCs increases cell proliferation, whereas TNC suppresses co-cultured BMSCs and TSCs proliferation. Furthermore, 1:1 BMSC/TSC co-culture and TNC treatment can lead to osteogenesis or chondrogenesis. ROCK, IGF-IR, and MEK might be involved in the TNC-induced differentiation of BMSCs and TSCs. ROCK increases TNC-induced osteocyte differentiation and decreases TNC-induced chondrocyte differentiation. IGF-1R and MEK upregulate TNC-induced osteogenesis and chondrogenesis. Although our study does not directly evaluate the resulting cells for their tendon-bone healing and regenerative ability, our results imply that the co-culture of BMSCs/TSCs at a 1:1 ratio combined with TNC treatment may be a potential approach to increase cell proliferation, which might be useful for tendon-bone healing.

## Abbreviations

BMSCs,: bone marrow-derived stem cells
TSCs,: tendon stem cells
TNC,: Tenascin C
ROCK,: Rho-associated kinase
IGF-1R,: insulin-like growth factor 1 receptor
MEK,: mitogen-activated protein kinase
BMP,: bone morphogenetic protein
FGF,: fibroblast growth factor
TGF-β,: transforming growth factor beta
VEGF,: vascular endothelial growth factor
CTGF,: connective tissue growth factor
PDGF,: platelet-derived growth factor
ECM,: extracellular matrix
PBS,: phosphate-buffered saline
DMEM,: Dulbecco’s modified Eagle’s medium
CCK-8,: cell counting kit-8
PPARγ,: peroxisome proliferators-activated receptor γ
RUNX2,: runt-related transcription factor 2
SCX,: Scleraxis
SOX-9,: Sry related HMG box gene 9
